# Starting from scratch: Step-by-step development of diagnostic tests for SARS-CoV-2 detection by RT-LAMP

**DOI:** 10.1371/journal.pone.0279681

**Published:** 2023-01-26

**Authors:** Diana Angélica Tapia-Sidas, Brenda Yazmín Vargas-Hernández, José Abrahán Ramírez-Pool, Leandro Alberto Núñez-Muñoz, Berenice Calderón-Pérez, Rogelio González-González, Luis Gabriel Brieba, Rosalía Lira-Carmona, Eduardo Ferat-Osorio, Constantino López-Macías, Roberto Ruiz-Medrano, Beatriz Xoconostle-Cázares

**Affiliations:** 1 Departamento de Biotecnología y Bioingeniería, Centro de Investigación y de Estudios Avanzados, Mexico City, Mexico; 2 Laboratorio Nacional de Genómica para la Biodiversidad, Centro de Investigación y de Estudios Avanzados, Irapuato, Guanajuato, Mexico; 3 Unidad de Investigación Médica en Enfermedades Infecciosas y Parasitarias, Hospital de Pediatría, Centro Médico Nacional Siglo XXI, Instituto Mexicano del Seguro Social, Mexico City, Mexico; 4 División de Investigación en Salud, UMAE Hospital de Especialidades “Dr. Bernardo Sepúlveda Gutiérrez”, Centro Médico Nacional Siglo XXI, Instituto Mexicano del Seguro Social, Mexico City, Mexico; Consejo Superior de Investigaciones Cientificas, SPAIN

## Abstract

The pandemic caused by the severe acute respiratory syndrome coronavirus 2 (SARS-CoV-2) has affected millions of people worldwide. Public health strategies to reduce viral transmission are based on widespread diagnostic testing to detect and isolate contagious patients. Several reverse transcription (RT)-PCR tests, along with other SARS-CoV-2 diagnostic assays, are available to attempt to cover the global demand. Loop-mediated isothermal amplification (LAMP) based methods have been established as rapid, accurate, point of care diagnostic tests for viral infections; hence, they represent an excellent alternative for SARS-CoV-2 detection. The aim of this study was to develop and describe molecular detection systems for SARS-CoV-2 based on RT-LAMP. Recombinant DNA polymerase from *Bacillus stearothermophilus* and thermostable engineered reverse transcriptase from Moloney Murine Leukemia Virus were expressed using a prokaryotic system and purified by fast protein liquid chromatography. These enzymes were used to set up fluorometric real time and colorimetric end-point RT-LAMP assays. Several reaction conditions were optimized such as reaction temperature, Tris-HCl concentration, and pH of the diagnostic tests. The key enzymes for RT-LAMP were purified and their enzymatic activity was determined. Standardized reaction conditions for both RT-LAMP assays were 65°C and a Tris-HCl-free buffer at pH 8.8. Colorimetric end-point RT-LAMP assay was successfully used for viral detection from clinical saliva samples with 100% sensitivity and 100% specificity compared to the results obtained by RT-qPCR based diagnostic protocols with Ct values until 30. The developed RT-LAMP diagnostic tests based on purified recombinant enzymes allowed a sensitive and specific detection of the nucleocapsid gene of SARS-CoV-2.

## Introduction

Coronavirus disease 2019 (COVID-19) is caused by a new highly infectious virus identified as severe acute respiratory syndrome coronavirus 2 (SARS-CoV-2). Due to the high spread of this virus worldwide, COVID-19 outbreak was declared a global pandemic on March 11^th^, 2020, by the World Health Organization [1]. SARS-CoV-2 belongs to *Betacoronavirus* genus, which members are known to cause respiratory diseases in humans and animals [2]. Symptoms of COVID-19 range from mild to severe. Mild infections can cause fever, chills, cough, fatigue, anosmia, dysgeusia, ageusia, dyspnea or difficulty breathing, among other symptoms [3]. In severe cases, SARS-CoV-2 can cause pneumonia, acute respiratory distress syndrome, organ failure, and can even lead to death [4]. High proportion of infected persons are asymptomatic (40 to 45%) and can transmit the virus for up to 14 days, accounting for an important source of virus propagation since they can go undetected without an appropriate diagnosis [5, 6]. One of the main strategies to monitor and control the viral dissemination has been the massive application of detection tests, which allow to identify and isolate infected individuals [7, 8]. SARS-CoV-2 detection has been accomplished mainly by two methods: immunoassays and nucleic acid amplification tests (NAAT). Real-time quantitative reverse transcription PCR (RT-qPCR) based methods have been widely used to address concern of disease diagnosis [9]. The "gold standard" test pioneered by the United States Centers for Disease Control and Prevention (CDC) for SARS-CoV-2 detection is sensitive and specific, although it is highly complex, expensive, and time-consuming. Furthermore, specialized reagents, equipment and personnel are required [10]. New strategies and protocols for viral detection are being developed and evaluated worldwide, with sights to improve their availability, accuracy, practicality and speed [11].

Reverse transcription loop-mediated isothermal amplification (RT-LAMP) is a nucleic acid detection technique that has become an excellent alternative for point-of-care (POC) testing because it is fast, simple, sensitive, and specific [12–14]. This method employs *Geobacillus stearothermophilus*, previously *Bacillus stearothermophilus* (Bst) DNA polymerase with high strand displacement activity that allows repeated amplification cycles without thermal denaturation. By combination with a thermostable reverse transcriptase, RNA sequences can be efficiently amplified under isothermal conditions [15]. The technique utilizes a set of four or six primers to recognize different regions of the target sequence, allowing an exponential amplification with high sensitivity and specificity [13, 16]. RT-LAMP amplification can be detected visually through colorimetric reactions. For instance, the use of pH-sensitive indicator dyes offers a rapid, robust, clear, and easy detection. This represents a feasible alternative for POC diagnostic assays [17]. In contrast, real-time fluorometric detection offers greater sensitivity, efficiency, precision, and the possibility of quantifying viral loads [18, 19]. Several studies have reported RT-LAMP assays for the detection of SARS-CoV-2 [20–25]. However, most of these use commercial reaction mixtures and enzymes subjected to availability that can limit diagnostic tests during the pandemic [26]. In this context, the objective of this research was to develop fast and practical RT-LAMP detection systems for SARS-CoV-2 with self-produced enzymes.

## Materials and methods

### RT-LAMP primer evaluation

RT-LAMP primers were used to target a conserved region of the nucleocapsid (N1) gene of SARS-CoV-2 (MW980115.1) [21] and the human ribonuclease (RNase) P (U94316.1) [27] as internal control. The primer sets included two inner primers (forward inner primer (FIP) and backward inner primer (BIP)), two outer primers (forward outer primer (F3) and backward outer primer (B3)), and two loop primers (forward loop primer (LF) and backward loop primer (LB)) (S1 Table). Evaluation of N1 primers targeting the viral region and the human RNase P (RP) primers was performed by LAMP assay using 1X WarmStart LAMP Master Mix (New England Biolabs, Beverly, MA, USA), 0.4 μM FIP/BIP, 0.2 μM F3/B3, 0.2 μM LF/LB N1 primers or 0.8 μM FIP/BIP, 0.2 μM F3/B3, 0.4 μM LF/LB RP primers, and 5 ng of pDrive-N1 plasmid (see below) or human total RNA control as template. LAMP reactions were incubated in a T100^TM^ ThermalCycler (Bio-Rad, Hercules, CA, USA) at 65°C for 40 min. Amplification products were visualized by electrophoresis through 2% agarose gel.

### DNA/RNA synthesis and reference material extraction for RT-LAMP

Three types of positive controls were used for RT-LAMP assays: plasmids, *in vitro* transcripts, and reference samples. For pDrive-N1 and pDrive-RP plasmid construction, RT-PCR of the gene fragments corresponding to N1 (202 bp) and RP (232 bp) was performed using the F3/B3 primers and a positive RNA sample from previously confirmed infected individual. SuperScript™ III Platinum™ One-Step qRT-PCR Kit (Thermo Scientific, Waltham, MA, USA) was used following the manufacturer’s instructions. The PCR products were cloned into the pDrive vector (Qiagen, Hilden, Germany) and verified by Sanger sequencing (Macrogen, South Korea). For *in vitro* transcripts, 9 μg of pDrive-N1 or pDrive-RP plasmid were linearized with BamHI-HF or HindIII-HF restriction enzymes (New England Biolabs, Beverly, MA, USA) respectively, and purified with Zymoclean Gel DNA Recovery Kit (Zymo Research, La Jolla, CA, USA). *In vitro* transcription was performed using 500 ng of linearized plasmid as template with the MAXIscript SP6/T7 Transcription Kit (Invitrogen, Waltham, MA, USA) according to manufacturer’s instructions. An extended incubation time of 1 h for DNase digestion was used. SP6 or T7 RNA polymerase was selected to produce transcripts with the same polarity as SARS-CoV-2 genome. Transcribed products were mixed with 25 μL of a lithium chloride solution (7.5 M LiCl, 10 mM EDTA) and incubated overnight at -20°C. RNA was recovered by centrifugation at 21,000 g for 45 min at 4°C. The pellet was washed with 300 μL of cold 70% ethanol, centrifugated at 21,000 g for 45 min at 4°C and resuspended in 30 μL of RNase-free water. The single stranded RNA (ssRNA) copy number was determined using Eq 1 [28]:

$$\text{ssRNA}\;\text{copy}\;\text{number}\;(\text{molecules})=\frac{X\;\text{ng}*6.022\times 10^{23}\text{molecules}/\text{mol}}{(N*340\;\text{g}/\text{mol})*1\times 10^{9}\;\text{ng}/\text{g}}$$ (1)

where *X* is the amount (ng) of ssRNA and *N* is the length (bp) of ssRNA. Ten-fold serial dilutions (from 5×10^10^ to 50 copies/μL) were prepared. For reference samples, 150 μL of AccuPlex SARS-CoV-2 Reference Material Kit (SeraCare, Milford, MA, USA), including reference materials and negative control, were extracted using the Viral RNA Purification Kit (Biopure, CDMX, Mexico), following the manufacturer’s instructions.

### Expression and purification of recombinant Bst and RT

Recombinant Bst DNA polymerase large fragment (U33536.1) and a quadruple mutant (E286R/E302K/L435R/D524A, designated as MM4) of the reverse transcriptase (RT) from Moloney Murine Leukemia Virus (MMLV) [29, 30] were cloned and expressed in *Escherichia coli* BL21 (DE3). A detailed protocol can be found at protocols.io for Bst (doi.org/10.17504/protocols.io.dm6gpb2e8lzp/v1) and RT (doi.org/10.17504/protocols.io.8epv59objg1b/v1) recombinant enzymes production. Chemically competent cells harboring pKJE7 plasmid (Takara Bio, San Jose, SA, USA) were obtained through rubidium chloride protocol [31]. BL21(DE3)/pKJE7 were transformed with pColdI-Bst or pET-MM4-RT expression vectors. Small-scale screening cultures to assess different conditions such as IPTG concentration (0.1, 0.5 or 1mM), induction temperature (16 or 37°C), and growth medium [Luria Bertani (LB) or terrific broth (TB)] were evaluated in triplicate for the recombinant protein expression. Carbenicillin (100 μg/mL) and chloramphenicol (30 μg/mL) were used as selective agents. For larger scale production, ten milliliters (10 mL) of overnight culture were added to 1 L of LB broth supplemented with carbenicillin (100 μg/mL) and chloramphenicol (30 μg/mL). Cell cultures were grown at 37°C at 200–220 rpm until an optical density at 600 nm (OD_600_) of 0.6 was reached, these were chilled on ice-water bath for 30 min and 0.5 mM IPTG was added for induction of recombinant proteins. No additional inducers were added as basal expression of chaperones contained in pKJE7 plasmid was considered appropriate to promote correct Bst and RT folding. Induced cell cultures were incubated at 16°C at 180 rpm for 16 h. After induction, cells were harvested by centrifugation at 6,000 g for 12 min at 4°C. The biomass was resuspended in 50 mL of lysis buffer A (LB-A: 50 mM Tris-HCl, pH 7.5, 0.5 mM EDTA, 10 mM 2-mercaptoethanol, 0.1% Tergitol NP-40, 0.1% Tween-20, 3 mM PMSF) for Bst or lysis buffer B (LB-B: 50 mM NaH_2_PO_4_/Na_2_HPO_4_, pH7.8, 300 mM NaCl, 2.5 mM DTT, 5% glycerol, 10 mM imidazole, 3 mM PMSF) for RT cell pellets. Cells were disrupted by ultrasonication in an ice bath using an UltraSonic Processor CPX750 (40% amplitude, 4 min: 10 s of ultrasonication per 10 s pause) (Cole Parmer, Vernon Hills, IL, USA). Lysates were centrifugated at 11,000 g for 30 min at 4°C. For heat clarification, Bst supernatant was incubated in a water bath at 60°C for 20 min and centrifugated at 11,000 g for 30 min at 4°C. RT supernatant and Bst clarified fractions were filtered through a 0.45 μm membrane. Immobilized metal affinity chromatography (Ni^2+^-IMAC), desalting, heparin affinity and cation exchange chromatography (CEC) were performed using an AKTA Pure 25 purification system (GE HealthCare, Chicago, IL, USA).

For Bst purification, the clarified fraction was diluted at a 1:1 ratio with saline buffer (SB: 50 mM Tris-HCl, pH 7.5, 100 mM NaCl, 1 mM PMSF) and loaded onto a HisTrap HP 5 mL column (GE Healthcare, Chicago, IL, USA) equilibrated with 8 column volumes (CV) of washing buffer-AI (WB-AI: 50 mM Tris-HCl, pH7.5, 100 mM NaCl, 10 mM imidazole, 1 mM PMSF). The column was washed with 10 CV of WB-AI, 10 CV of 2% elution buffer-AI (EB-AI: 50mM Tris-HCl, pH7.5, 100 mM NaCl, 500 mM imidazole, 1 mM PMSF) and eluted with 5 CV of 100% EB-AI. Fractions were pooled and desalted with desalting buffer-A (DB-A: 50 mM Tris-HCl, pH 7.5, 20 mM KCl, 1 mM EDTA, 1 mM DTT, 1mM PMSF) using HiPrep 26/10 Desalting Column (GE Healthcare, Chicago, IL, USA). Desalted fractions were identified by qualitative Bradford assay using the QuickStart Bradford 1X Dye Reagent (Bio-Rad, Hercules, CA, USA). They were loaded onto an HiTrap Heparin HP 5 mL column (GE Healthcare, Chicago, IL, USA) equilibrated with 10 CV of washing buffer-AII (WB-AII: 50 mM Tris-HCl, pH7.5, 1 mM EDTA, 1 mM DTT, 1 mM PMSF) and washed with 5 CV of WB-AII. Bounded proteins were eluted with a linear gradient of 10 CV of elution buffer-AII (EB-AII: 50 mM Tris-HCl, pH7.5, 1 M KCl, 1 mM EDTA, 1 mM DTT, 1 mM PMSF). The fractions containing the purified Bst enzyme were pooled and dialyzed overnight at 4°C in a 1:50 (v/v) ratio against storage buffer-A (SB-A: 10 mM Tris-HCl, pH 7.5, 50 mM KCl, 2 mM DTT, 0.1 mM EDTA, 50% glycerol). Finally, Bst was concentrated using an Amicon Ultra-15ML—30 kDa cut-off centrifugal filter (Merck, Kenilworth, NJ, USA), 0.1% triton X-100 was added, and enzyme aliquots were stored at -20°C.

RT purification was similar to Bst, with several changes indicated as follows. Ni^2+^-IMAC column was equilibrated and washed with LB-B. Second washing and elution steps were performed using elution buffer-BI (EB-BI: 50 mM NaH_2_PO_4_/Na_2_HPO_4_, pH7.8, 300 mM NaCl, 2.5 mM DTT, 5% glycerol, 500 mM imidazole, 1 mM PMSF), and 2 mM EDTA and 2.5 mM DTT were added to the eluted fractions. Desalting was performed using desalting buffer-B (DB-B: 50 mM HEPES, pH 7.5, 40 mM NaCl, 2 mM EDTA, 5 mM DTT, 5% v/v glycerol, 1 mM PMSF). For the second purification step, CEC was performed using HiTrap SP XL 5 mL column (GE Healthcare, Chicago, IL, USA) equilibrated and washed with DB-B. Elution buffer-BII (EB-BII: 50 mM HEPES, pH 7.5, 1M NaCl, 2 mM EDTA, 5 mM DTT, 5% v/v glycerol, 1 mM PMSF) was used for CEC elution step applying a linear gradient. RT fractions were dialyzed against storage buffer-B (SB-B: 50 mM Tris-HCl, pH 7.5, 150 mM NaCl, 1 mM DTT, 0.1 mM EDTA, 50% v/v glycerol). In the final step, 0.05% NP-40 was used, and enzyme aliquots were stored at -20°C.

All chromatographic steps were analyzed by electrophoresis through 8% tricine-SDS-PAGE gels. Bst and RT protein concentration was determined using a NanoDrop One Spectrophotometer (Thermo Scientific, Waltham, MA, USA) and purity was evaluated by densitometry of Coomassie brilliant blue stained tricine-SDS-PAGE gels using the Image Lab 6.1 software (Bio-Rad, Hercules, CA, USA).

### Bst and RT enzymatic activity assays

Different concentrations of recombinant Bst and RT were obtained through serial dilutions in SB-A or SB-B, respectively. To evaluate Bst enzymatic activity, DNA amplification by LAMP assays was performed in 12.5 μL final volume reactions containing 1X Isothermal Amplification Buffer (New England Biolabs, Beverly, MA, USA), 1.4 mM dNTPs, 6 mM MgSO_4_, 40 mM guanidine hydrochloride (GuHCl), 1.6 μM FIP/BIP, 0.2 μM F3/B3, 0.4 μM LF/LB, 437.5 or 43.75 pg of pDrive-N1 as template, and 1072, 536, 432, 320, 272, 136 or 64 ng of recombinant Bst. LAMP reactions were incubated at 65°C for 30 min. To evaluate recombinant RT enzymatic activity, cDNA synthesis was performed with the SuperScript III™ First-Strand Synthesis System reagents (Invitrogen, Waltham, MA, USA) following the manufacturer’s instructions. Reaction mixtures contained 0.5 μM F3/B3 RP primers, 40 ng/μL of *in vitro* RP transcript and 8, 40, 200 or 420 ng of recombinant RT. All reactions were evaluated by electrophoresis in 2% agarose gel, and nucleic acids produced amounts were determined by densitometric analysis using the Image Lab 6.1 software (Bio-Rad, Hercules, CA, USA) using 1 μL of 1 Kb Plus DNA Ladder (Invitrogen, Waltham, MA, USA) as standard. The total amount of nucleic acid produced was plotted against the total amount of enzyme per reaction. Then, the equation of the exponential region of the curve was obtained and used to determine the specific enzymatic activity. Bst and RT specific enzymatic activity was expressed as units per milligram (U/mg) of enzyme. One unit of Bst was defined as the amount (ng) of enzyme required to produce 100 ng of dsDNA in 30 min at 65°C, while one unit of RT was defined as the amount (ng) of enzyme required to produce 100 ng of cDNA in 60 min at 50°C.

### Optimization assays of RT-LAMP for SARS-CoV-2 detection

The amplification temperature (60, 63 and 65°C) was evaluated in triplicate by fluorometric real time LAMP assays. Reaction mixtures contained 1X reaction buffer [50 mM KCl, 10 mM (NH_4_)_2_SO_4_, 2 mM MgSO_4_, and 0.1% Triton X-100], 1.4 mM dNTPs, 1X SYBR Green I (Rx Biosciences, Gaithersburg, MD, USA), 520 ng of recombinant Bst and 100 pg of pDrive-N1 as template. We evaluated Tris-HCl (0, 0.4, 0.8, 1.0 mM) and pH (8.5 or 8.8) effects in triplicate by real time RT-LAMP reactions using 280 ng of recombinant RT and 1×10^6^ copies of N1 *in vitro* transcript as template. Subsequently, the effect of pH 8.8 buffer without Tris-HCl in colorimetric end point RT-LAMP was evaluated in duplicate. Each 12.5 μL final volume reaction contained 1X colorimetric reaction buffer [40 mM K_2_SO_4_, 1 mM (NH_4_)_2_SO_4_, 2 mM MgSO_4_, 0.1% Triton X-100, 200 μM phenol red], 1.4 mM dNTPs, 0.4 μM FIP/BIP, 0.2 μM F3/B3, 0.2 μM LF/LB, 280 ng of recombinant RT, 520 ng of recombinant Bst and 1×10^5^ copies of N1 *in vitro* transcript. All real-time RT-LAMP reactions were performed in a StepOnePlus^TM^ Real Time PCR System (Applied Biosystems, Foster City, CA, USA) with a program of 30 or 40 cycling stage of 1 min at 65°C, with fluorescence reading at the end of each cycle. Colorimetric end-point RT-LAMP reactions were performed in a T100^TM^ ThermalCycler (Bio-Rad, Hercules, CA, USA) at 65°C for 40 min. The reaction tubes were chilled on ice and color changes were documented. Amplification products were visualized by electrophoresis through 2% agarose gel. The effect of additives (40 mM GuHCl or 0.8 M betaine) was evaluated by fluorometric real-time and colorimetric end-point RT-LAMP assays as previously described using *in vitro* transcribed N1 gene fragment as template.

### Analytical sensitivity and specificity of RT-LAMP assays for SARS-CoV-2 detection

The analytical sensitivity or limit of detection (LOD) and specificity of RT-LAMP assays were evaluated in triplicate under optimized conditions. LOD was determined with 1×10^11^ to 1×10^2^ copies/reaction of N1 *in vitro* transcript. The analytical specificity was determined by fluorometric real-time RT-LAMP assay using the NATtrol Respiratory Panel-2 (RP2) Controls, control 1 (NATROL-1) and control 2 (NATROL-2) (ZeptoMetrix, Buffalo, NY). AccuPlex SARS-CoV-2 reference material (SeraCare, Milford, MA, USA) and 1×10^9^ copies of *in vitro* transcribed N1 gene fragment were used as positive controls.

### Evaluation of the RT-LAMP assays for SARS-CoV-2 detection in clinical samples

The study was conducted according to the guidelines of the Declaration of Helsinki and approved by the Ethics Committee for Human Research of Centro de Investigación y de Estudios Avanzados (CINVESTAV) (Protocol number: 062/2020). Written, informed consent was signed by each participant. No minor participants were included in this study.

Saliva samples were collected from 100 volunteers from people seeking testing for SARS-CoV-2 at CINVESTAV, Mexico, in September 2021 employing the Biopure Self-Sampling Kit (Biopure, CDMX, Mexico) and following the manufacturer’s instructions. Collected samples were inactivated at 65°C for 20 min. RNA extraction was performed using Viral RNA Purification Kit (Biopure, CDMX, Mexico) and quantified using a NanoDrop One (Thermo Scientific, Waltham, MA, USA). Purified RNA samples were analyzed by colorimetric end-point RT-LAMP assays. Reactions of 12.5 μL were performed with recombinant enzymes, without additives, and optimized conditions with N1 primers and RP primers. Additionally, samples were evaluated by RT-qPCR according to two different diagnostic methodologies: CDC and Berlin protocols. For CDC RT-qPCR protocol, 2019-nCoV-N1 (target: a region of the N gene) and RP (target: human RNase P) primers/probe sets were used [32]. For Berlin protocol, RdRp_SARSr (target: RNA-dependent RNA polymerase viral gene) forward and reverse primers, with RdRp_SARSr-P1 (for SARS-CoV-2, SARS-CoV and bat-SARS-related CoVs detection) or RdRp_SARSr-P2 (specific for SARS-CoV-2, will not detect SARS-CoV) probes were used [33].

### Statistical analysis

To determine significant statistical differences between treatments, non-parametric analysis Kruskall-Wallis was employed to compare the means of groups. When significant differences were observed, Dunn’s multiple comparisons test was performed to determine which groups were different. For real time RT-LAMP fluorometric data, we compare the time to threshold to determine statistical significance. Means and standard deviations (SD) were calculated and represented as mean ± SD. All calculations and graphics were performed with the GraphPad Prism software version 8.0.1 by setting a p < 0.05 as a significant value.

## Results

### RT-LAMP primer evaluation

N1 primers target the nucleocapsid gene of SARS-CoV-2 that included a region of 202 bp from 28,239 to 28,440 of SARS-CoV-2 genome. The amplification performance of the RT-LAMP N1 and RP primer sets was evaluated by LAMP assays. Electrophoretic profile of the resulting products showed the typical ladder-like pattern, confirming the functionality of the primers at the concentrations employed (0.4 μM FIP/BIP, 0.2 μM F3/B3, 0.2 μM LF/LB of N1 primers and 0.8 μM FIP/BIP, 0.2 μM F3/B3, 0.4 μM LF/LB of RP primers) (S1 Fig).

### Expression and purification of recombinant Bst and RT

**Fig 1:**
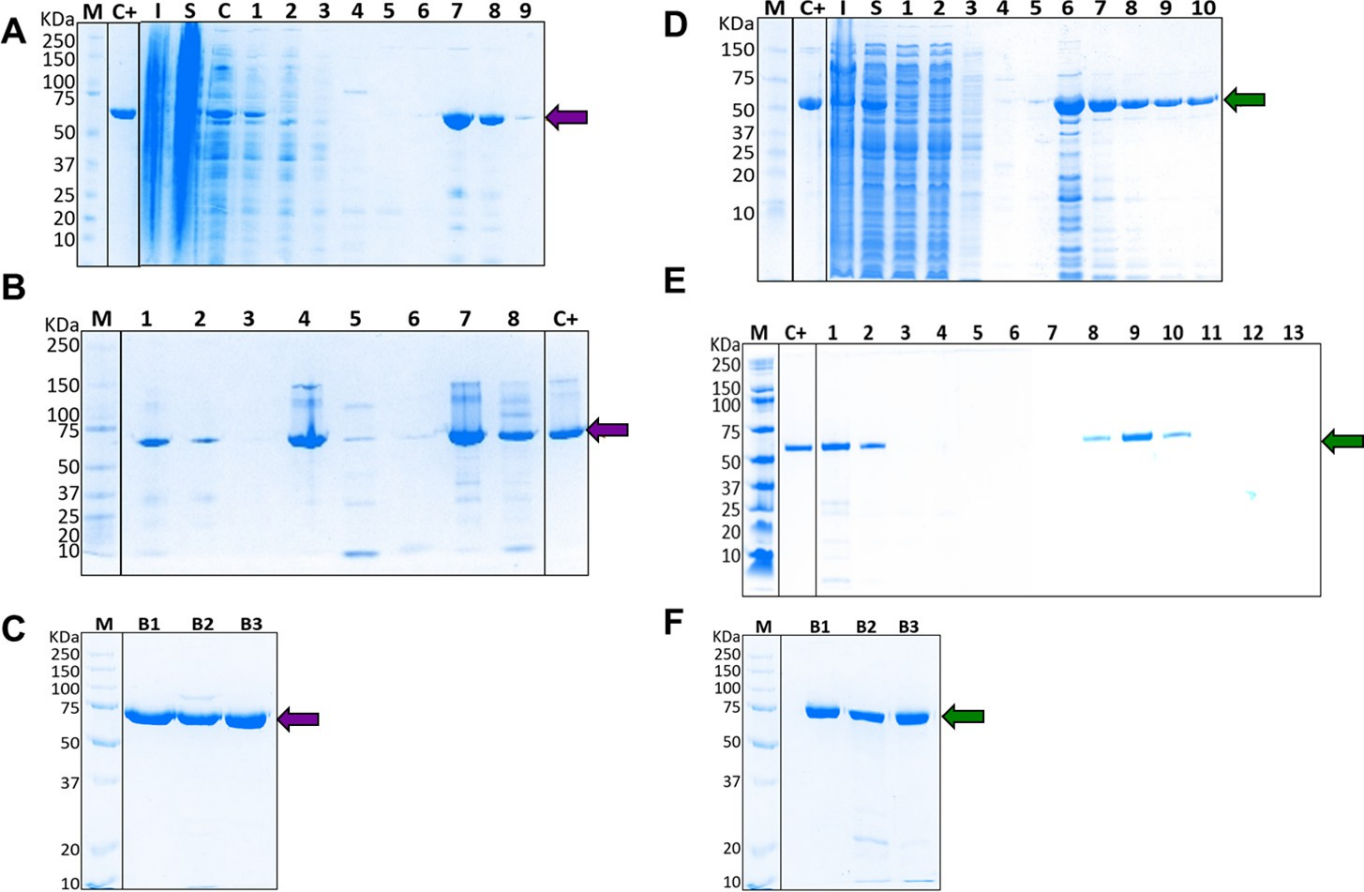
Expression and purification of recombinant *Bacillus stearothermophilus* DNA polymerase (Bst) and reverse transcriptase (RT) from Moloney Murine Leukemia Virus. Coomassie blue-stained 8% Tricine-SDS-PAGE electrophoresis gel analysis. (A) Expression of recombinant Bst enzyme and Ni^2+^-IMAC purification. Lane 1: clarified supernatant loaded; Lane 2: flow-through fraction; Lane 3–6: washing step fractions; Lane 7–9: eluted fractions containing Bst. (B) Heparin column purification of recombinant Bst. Lane 1: desalted sample loaded; Lane 2: flow-through fraction; Lane 3: washing step fraction; Lane 4–6: elution fractions; Lane 7–8: concentrated Bst-containing fractions. (C) Final Bst formulations from three different purification batches (B1, B2, B3). (D) Expression of recombinant RT enzyme and Ni^2+^-IMAC purification. Lane 1–3: flow-through fractions; Lane 4–5: washing step fractions; Lane 6–10: elution fractions containing RT. (E) Cation exchange column purification of recombinant RT. Lane 1: IMAC elution fraction; Lane 2: desalted sample loaded; Lane 3–5: flow-through fractions; Lane 6–7: washing step fractions; Lane 8–13: elution fractions. (F) Final RT formulations from three different purification batches (B1, B2, B3). M: molecular weight marker; C+: previously purified Bst or RT enzyme, employed as control positive; I: insoluble fraction; S: soluble fraction; C: clarified supernatant. Violet or green arrows indicate the expected size for Bst and RT enzymes, respectively.

For recombinant protein production in *E*. *coli* carrying the pKJE7 and Bst or RT plasmids, induction conditions were selected to obtain the maximum yield of soluble protein (S2 and S3 Figs). Both enzymes were induced at 16°C for 16 h in LB medium with 0.5 mM IPTG, recovered in the soluble fraction, and purified by Ni^2+^-IMAC chromatography (Fig 1). The biomass yield of the recombinant expression cultures for the Bst and RT enzymes were 3.89 and 4.06 g/L, respectively. After the elution with 500 mM imidazole, recombinant Bst and RT were recovered in two and five fractions, respectively (Fig 1A and 1D). The Ni^2+^-IMAC elution fractions were desalted and a second purification step through heparin chromatography (HC) for Bst enzyme (Fig 1B) and cation exchange chromatography (CEC) for RT (Fig 1E) were performed. All chromatographic steps showed a single peak corresponding to the fractions containing recombinant proteins (S4 Fig). SDS-PAGE analysis of HC for Bst revealed the enzyme was obtained in at least one of the eluted fractions (Fig 1B, Lane 4). Similarly, the second purification step for RT allowed to recover this enzyme in three elution fractions (Fig 1E, Lane 8–10). Densitometric analysis of SDS-PAGE final formulated fractions showed that recombinant Bst was obtained at 1.6–2.0 mg/mL (purity >85%) with an estimated size of 66.6 kDa (Fig 1C). On the other hand, recombinant RT was obtained at 1.2–1.5 mg/mL (purity ranging from 80.1% to 99.2%) with an estimated size of 72 kDa (Fig 1F).

### Bst and RT enzymatic activity assay

For Bst enzymatic activity assay evaluated by LAMP reactions, similar amounts of DNA were amplified with 1,072 ng and 536 ng of Bst. However, the amount of amplified DNA showed a slight decrease using 432 ng and a large decrease with 320 ng of recombinant Bst. Similar results were observed for both DNA plasmid concentrations used as template. For RT enzymatic activity assay evaluated by cDNA synthesis, 91 ng of cDNA were obtained with 420 ng and 200 ng of recombinant RT, while 96 ng of cDNA were obtained with 200 U of the commercial enzyme (SuperScript III RT, Invitrogen). The amount of synthesized cDNA decreased using 40 ng of recombinant RT and it was not detectable when 8 ng of RT were used (S5 Fig). The calculated specific enzymatic activity was 2.99×10^3^ U/mg for recombinant Bst and 4.15×10^3^ U/mg for RT.

### Optimization assays of RT-LAMP for SARS-CoV-2 detection

**Fig 2:**
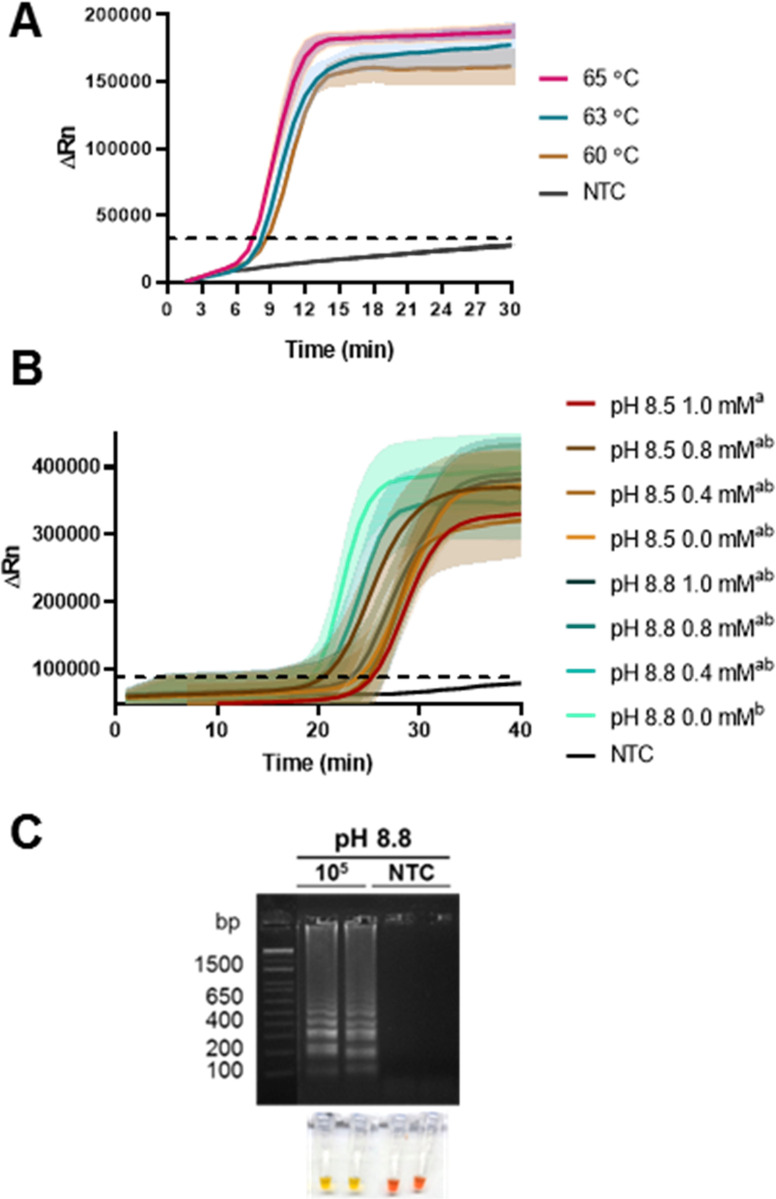
Optimization assays of RT-LAMP reaction conditions for SARS-CoV-2 detection. (A) Real time LAMP reactions evaluating the effect of temperature on amplification. (B) Real time RT-LAMP reactions at different pH and concentration of Tris-HCl. (C) Colorimetric RT-LAMP reactions at pH 8.8 in the absence of Tris-HCl in the reaction buffer. The figure shows the electrophoretic profile of the amplification reaction products (upper panel) and the colorimetric determination of each reaction (lower panel). NTC: non-template control; M: DNA molecular weight maker 1 Kb Plus DNA Ladder (Invitrogen). Shadows represent standard deviation from triplicate curves. Different lowercase letters (a, b) indicate significant differences among treatments based on Dunn’s test at p < 0.05.

To evaluate temperature effect, fluorometric real-time LAMP reactions were also performed. The N1 primer set showed a more efficient amplification at 65°C with statistically significant difference (Fig 2A). Subsequently, real-time RT-LAMP assays under different Tris-HCl concentrations (0 to 1 mM) and two pH values (8.5 and 8.8) were performed (Fig 2B). Tris-HCl concentration at different pH had little effect on the amplification of *in vitro* transcribed N1 RNA fragment, since only 1 mM Tris-HCl, pH 8.5 and absence of Tris in a pH 8.8 buffer showed statistically significant differences between treatments. For this reason, reaction buffer without Tris-HCl at pH 8.8 was selected as optimal for the fluorometric RT-LAMP assay (Fig 2B). On the other hand, the selected conditions were tested in colorimetric end-point RT-LAMP reactions. In absence of Tris-HCl (pH 8.8) an evident visual detection of color transition from pink-red (negative reaction) to yellow (positive reaction) was obtained. These conditions were also established as the optimal for the colorimetric RT-LAMP assay (Fig 2C).

### Effect of additives on RT-LAMP assays for SARS-CoV-2 detection

Fluorometric real time RT-LAMP reactions incorporating 40 mM GuHCl showed reduced Ct (cycle threshold) value (Ct mean = 12.1±0.4) compared with Ct of additive-free reactions (Ct mean = 14.9±0.3) or including 0.8 M betaine (Ct mean = 14.0±0.5), with statistically significant difference for GuHCl reactions. Nevertheless, colorimetric end-point RT-LAMP assays with 40 mM GuHCl exhibited color transition (yellow) in NTC reactions (false positives) (S6 Fig). For these reasons, GuHCl or betaine were not included as additives on RT-LAMP assays.

### Analytical sensitivity and specificity of RT-LAMP assays for SARS-CoV-2 detection

**Fig 3:**
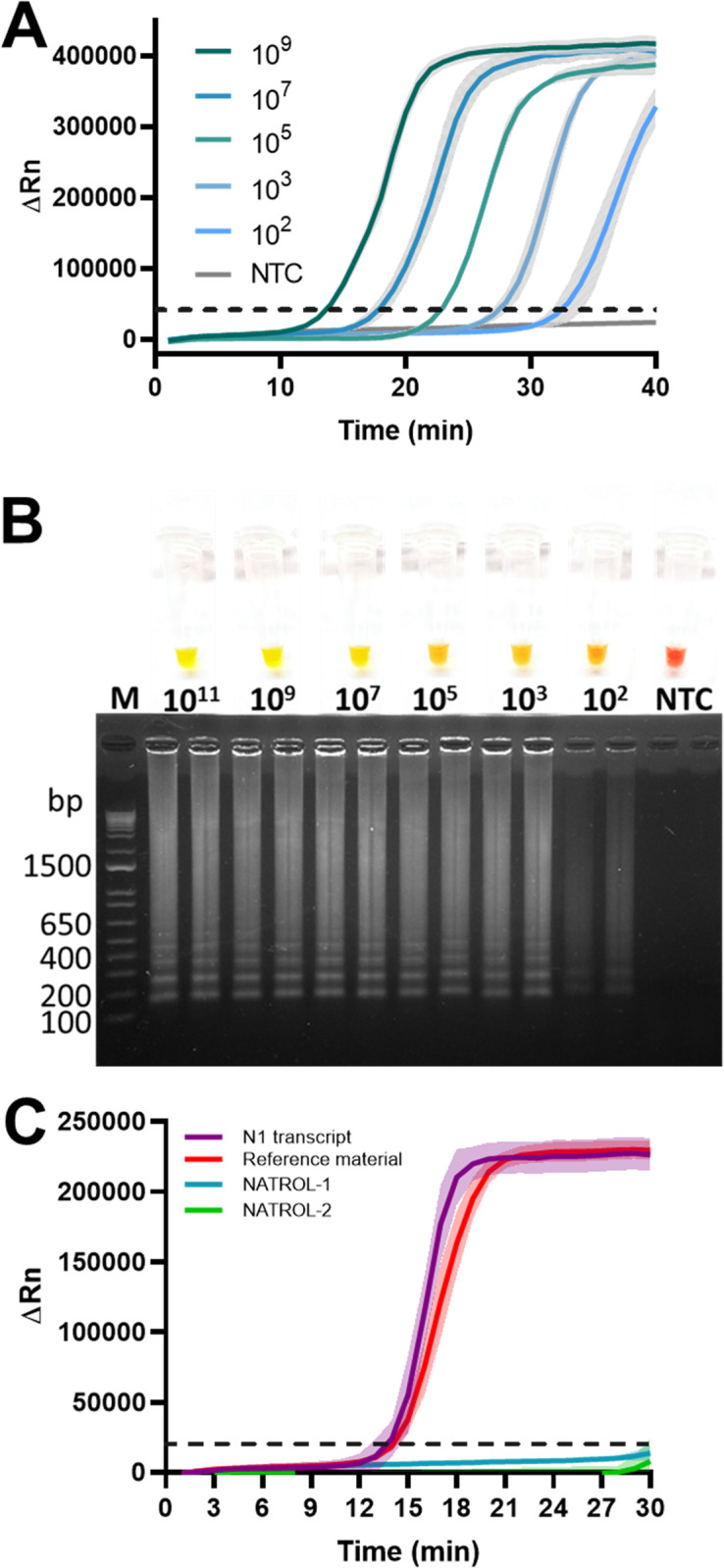
Analytical sensitivity and specificity of RT-LAMP. (A) Real time RT-LAMP amplification plots obtained from ten-fold serial dilutions of *in vitro* N1 transcript ranging between 10^9^ and 10^2^ copies/reaction. (B) Colorimetric change and electrophoretic amplifications obtained from ten-fold serial dilutions of *in vitro* N1 transcript ranging from 10^11^ to 10^2^ copies/reaction. (C) Evaluation of specificity through the optimized fluorometric real-time RT-LAMP assay with the recombinant Bst and RT enzymes against respiratory controls NATROL-1 and NATROL-2. NTC: non-template control. M: DNA molecular weight marker 1 Kb Plus DNA Ladder (Invitrogen). N1 transcript: 1×10^9^ copies of *in vitro* transcribed N1 gene. Reference material: 2 μL of AccuPlex SARS-CoV-2 reference material.

The analytical sensitivity of fluorometric real time and colorimetric end-point RT-LAMP assays was evaluated under optimized conditions. Amplification curves of serial dilutions of *in vitro* N1 transcript showed that fluorometric real time RT-LAMP assay was able to detect 10^2^ copies/reaction (Fig 3A). Analytical sensitivity analysis of colorimetric end-point RT-LAMP showed evident change color until 1x10^2^ copies/reaction (Fig 3B). For analytical specificity, respiratory controls (NATROL-1 and NATROL-2) were evaluated by fluorometric real time RT-LAMP without evident amplification (Fig 3C).

### Evaluation of the RT-LAM-P assays for SARS-CoV-2 detection in clinical samples

**Fig 4:**
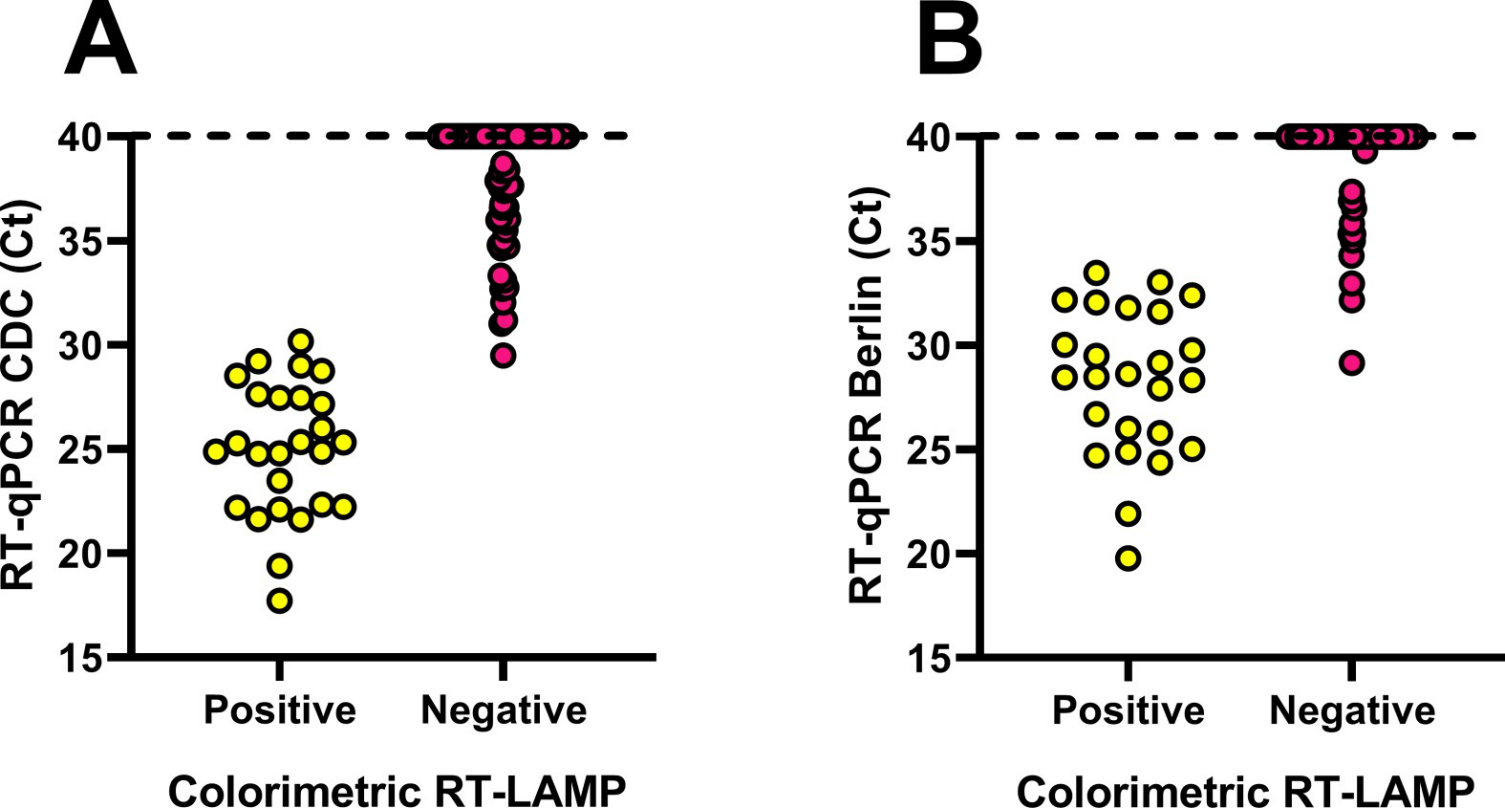
Evaluation of colorimetric end-point RT-LAMP assays for SARS-CoV-2 detection in clinical samples. RNA was extracted from saliva samples and evaluated for SARS-CoV-2 detection. Comparison of Ct values from RT-qPCR protocols and colorimetric end-point RT-LAMP (Colorimetric RT-LAMP) results from 100 samples tested. Negative samples (Ct > 40 and undetermined) for SARS-CoV-2 detection by RT-qPCR are shown at Ct = 40. (A) 59 samples were called positive by CDC RT-qPCR protocol-based methodology using 2019-nCoV-N1 primers/probe set (y-axis) and were compared to colorimetric RT-LAMP results (x-axis) detected as positive (yellow) or negative (pink). (B) 34 samples were called positive by Berlin RT-qPCR protocol-based methodology using RdRp_SARSr-P2 probe (y-axis) and were compared to colorimetric RT-LAMP results (x-axis) detected as positive (yellow) or negative (pink). Dashed lines at the top of each panel serve as reference for samples below the detection limit for RT-qPCR.

Finally, considering that colorimetric end-point RT-LAMP is a feasible alternative for POC diagnostics without the need for specialized equipment, we compared the performance of this assay with CDC and Berlin protocol-based methodologies (RT-qPCR). RNA extraction from 100 saliva samples from volunteers was performed. According to RP primers/probe set from CDC RT-qPCR protocol, internal control was detected in all samples with Ct values ranging from 24.90–31.93, and 59% were positive to N gene detection with Ct values of 17.71–38.70 (S1 File). Moreover, Berlin RT-qPCR protocol-based methodology using RdRp_SARSr-P2 probe resulted in 34% positive detection of SARS-CoV-2 with Ct values ranging from 16.68–37.16, and 38% positive detection of SARS-CoV-2, SARS-CoV and bat-SARS-related CoVs (Ct values from 19.77–39.31) using RdRp_SARSr-P1 probe in saliva samples. On the other hand, colorimetric end-point RT-LAMP resulted in 26% positive detection for SARS-CoV-2. Comparing RT-LAMP assay to RT-qPCR Ct values, sensitivity (defined as the ability of a screening test to detect a positive result, being based on the positive result rate [34] detected by a reference method) of RT-LAMP compared with CDC protocol-based Ct values below 30 was 100% (Table 1). However, RT-LAMP sensitivity decreased in samples with Ct values until 35 (68.4%) and performed poorly (44.1% sensitivity) in samples with Ct > 35. Out of 41 negative CDC results, none were observed to show a positive reaction when analyzed with RT-LAMP. Hence a 100% specificity (defined as the ability of a screening test to detect a negative result, being based on the negative result rate [34] detected by a reference method) was obtained for colorimetric RT-LAMP. Targeting SARS-CoV-2, the sensitivity of RT-LAMP compared with Berlin protocol-based Ct values until 30 was 100% but decreased to 81.3% and 76.5% for Ct > 30 and Ct > 35, respectively. Out of 66 Berlin protocol-based negative results, none showed a positive RT-LAMP reaction. Therefore, 100% specificity was obtained for colorimetric assay. These results suggested that colorimetric RT-LAMP assay developed with recombinant enzymes can be used for a sensitive and specific detection of SARS-CoV-2 in complex samples such as RNA purified from saliva (Fig 4).

**Table 1:** Comparison of SARS-CoV-2 detection by RT-LAMP and RT-qPCR (CDC and Berlin protocol-based methodologies) in saliva samples.

RT-qPCR			Colorimetric RT-LAMP		
Protocol-based methodology	Ct	n	Positives	Negatives	Sensitivity (%)*
**CDC (2019-nCoV-N1 primers/probe set)**	15–30	25	25	0	100.0
	15–35	38	26	12	68.4
	15–40	59	26	33	44.1
	Negative	41	0	41	-
Berlin (RdRp_SARSr-P2 probe)**	15–30	24	24	0	100.0
	15–35	32	26	6	81.3
	15–40	34	26	8	76.5
	Negative	66	0	66	-

*Sensitivity (%) = (RT-LAMP positives/RT-qPCR positives) x 100

**Specific for SARS-CoV-2

n: number of samples

## Discussion

The first step in managing COVID-19 is the rapid and accurate detection of SARS-CoV-2 enabled by identification of viral targets [35]. RT-LAMP is a nucleic acid detection technique that has become an excellent alternative for POC testing due to its characteristics of a rapid, simple, sensitive, and specific method [12–14]. Nevertheless, most of the reported assays for the detection of SARS-CoV-2 by RT-LAMP use commercial enzymes or reaction buffers subject to availability. In this work, RT-LAMP diagnostic tests (fluorometric real time assay and colorimetric end-point assay) were developed with emphasis in the production and purification of the enzymes necessary to implement the technique. For this purpose, Bst DNA polymerase and MM4-RT were expressed and purified. Previous studies have reported a high catalytic activity and thermostability for both enzymes [30, 36] which make them suitable to be used in RT-LAMP assays. Additionally, attending the high demand for diagnostic testing against SARS-CoV-2, we do not discard the possibility of using plasmids encoding for other DNA polymerases (Bsm, Bst2.0, Bst3.0 or Bst LF) or thermostable reverse transcriptases such as AMV or HIV retrotranscriptases [37, 38].

During the purification of the Bst enzyme, the heat clarification step allowed the elimination of a high proportion of non-thermostable proteins from *E*. *coli* [39], this step favored protein-resin interaction and protein recovery in the first purification by Ni^2+^-IMAC column. The second step by heparin chromatography, commonly used for the purification of proteins with affinity to nucleic acids [40], allowed the recovery of the active enzyme with high purity. Similarly, the purification process used for the RT enzyme allowed the protein to be recovered with high purity. The purification workflow proposed in this work for Bst and RT could be used to produce enzymes for the development of RT-LAMP-based detection tests.

RT-LAMP assays included six primers targeting SARS-CoV-2 nucleocapsid gene sequence. ORFs located in the 3’ region of viral genomes are usually better candidates for the development of detection tests against single-stranded RNA viruses, such as SARS-CoV-2 [41, 42]. Additionally, the 5’ region of N1 gene overlaps with ORF9b, which explains high conservation and low mutation rate [43, 44]. Nucleocapsid gene is present in the genomic RNA and in nine of the subgenomic RNA molecules of SARS-CoV-2, inferring the virus could be detected in all these molecules (Fig 1A). Bioinformatic analysis demonstrated that the primers matched 100% the N gene of the alpha, beta and delta SARS-CoV-2 variants of concern, while one nucleotide mismatch was found for the omicron variant. This suggests that RT-LAMP assays have a broad capacity to accurately detect several SARS-CoV-2 variants. Primer concentration is critical for RT-LAMP reactions as an inadequate amount could decrease the sensitivity of the assay, and consequently could affect the reliability of the detection test [45]. In this study, a low primer concentration for N1 and RP primer sets showed an appropriate performance as no by-product formation was observed and the amplification efficiency of the target gene was not affected.

A decrease in the total ion concentration (Tris-HCl, KCl, MgCl_2_) has been shown to affect reaction efficiency [46]. Our results indicated that buffer composition for fluorometric real-time and colorimetric end-point RT-LAMP assays was adequate, as optimal enzymatic activity, and a high reaction efficiency were obtained. Both assays could be carried out in absence of Tris-HCl pH 8.8 without affecting the reaction, even the color transition in the case of colorimetric detection method. Although it has been reported that additives such as guanidine hydrochloride (GuHCl) [47, 48] and betaine [45, 49] can improve the speed and sensitivity of RT-LAMP reactions. However, we did not detect statistical differences after betaine addition and GuHCl interfered with visual detection. Previous reports indicated variable and stochastic results with RT-LAMP assays when less than 100 copies of synthetic viral RNA standard per reaction were used [37, 47]. Instead, a LOD equal or higher than 1000 copies per reaction resulted in a robust detection of the target gene [37]. Therefore, considering the LOD obtained in this work, it would be expected a sensitive and robust detection of SARS-CoV-2 by both developed RT-LAMP assays. A common strategy to achieve lower LOD than those obtained here is to use two or more target genes in the same reaction [50]. In addition, the detection of SARS-CoV-2 N gene by RT-LAMP using the selected primers showed to be highly specific as no cross-reactivity was observed with several common respiratory pathogens including influenza virus isolates (AH1, AH1N1, AH3 and B), parainfluenza (type 1, 1A, 2 and 4), adenovirus (type 1, 3 and 31), *Mycoplasma pneumoniae*, human Metapneumovirus, *Legionella pneumophila*, *Chlamydophyla pneumoniae*, *Bordetella pertussi*s, *Bordetella parapertussis*, RSV type A, respiratory syncytial virus A, and other coronaviruses (NL63, OC43, HKU-1, 229E).

The developed colorimetric RT-LAMP assay was subjected to a clinical evaluation for SARS-CoV-2 detection in saliva samples. A high rate of false positives has been reported in colorimetric RT-LAMP assays using phenol red caused by the high pH variability of saliva samples. Indeed, the pH range of saliva (6.8 to 7.4) is close to the pH transition zone of phenol red (pH 7.5) [17, 51, 52]. We employed saliva instead of conventional nasopharyngeal swabs samples since discomfort, coughing and sneezing are usually produced during sample collection [53]. In addition, this stage can represent a critical moment of exposure to the virus in healthcare professionals [54]. Saliva samples possess high heterogenicity [55, 56], low yields during RNA extraction [56, 57], presence of polymerase inhibitors [58] and genetic material from the oral microbiome [59]. However, RT-LAMP is more resistant to inhibitors than RT-qPCR [60], and saliva samples are especially useful for screening asymptomatic patients or those with mild COVID-19 infection [61]. Furthermore, it has recently been described as the ideal biological fluid for the detection of strains with tropism towards the upper respiratory tract (such as the omicron variant) [62].

Developed colorimetric end-point RT-LAMP assay using the recombinant Bst and RT enzymes was able to amplify the human RNase P gene in all samples that were positive for this internal reaction control by CDC RT-qPCR test (S1 File). Considering previous reports of different RT-LAMP procedures compared with several RT-qPCR reference protocols, sensitivity for SARS-CoV-2 detection is nearly 100% for Ct values until 30 using distinct viral targets in pharyngeal swab samples [63, 64]. Ct values close to 30 have been reported as cut-off in RT-qPCR assays compared with LAMP for the detection of SARS-CoV-2 [20, 65] due to they are related with low viral load samples and they are close to the detection limit for viral culture with probably scarce epidemiological significance [66–68]. Other studies have shown that Ct values > 30 are useful predictors of low infectivity, low risk of intubation, and low mortality [69, 70], and this has been used as hospital discharge criteria [71, 72]. Indeed, detection of SARS-CoV-2 by colorimetric end-point RT-LAMP assay developed in this work demonstrated a 100% sensitivity among samples with Ct < 30 when RT-qPCR CDC or Berlin diagnostic methodologies were used as reference protocols. However, considering CDC and Berlin Ct values up to 35 and 40, sensitivity of our developed assay decreased. It has been reported that clinical samples with Ct values > 30 (corresponding to low viral load) made the detection process quite challenging [73]. Even RT-qPCR assays could detect reliably specimens with Ct values < 30, but did not detect 40–60% of specimens with Ct ≥ 30 [73]. In this regard, we observed a discrepancy between CDC and Berlin positive results (59% and 34%, respectively) using RNA extracted from saliva samples (Table 1). According to a previous study, four WHO approved RT-PCR diagnostic protocols for SARS-CoV-2 (including those used in our study) showed discordant results in almost 30% of cases evaluated [74]. Additionally, Berlin RdRp-SARSr primers-probe set has been reported to show low sensitivity compared with other primers-probe sets [75]. Finally, 100% specificity was obtained for our colorimetric end-point RT-LAMP assay since no false positive reactions were observed in comparison with RT-qPCR CDC or Berlin reference protocols.

The main advantage of our method is that it is fully independent on commercial suppliers (enzymes or buffers), that could be subjected to limited availability due to the ongoing pandemic and necessity of continuous diagnostic test. Our development of RT-LAMP assays using recombinant enzymes and in-house-made buffers, together with other efforts [76, 77], confirmed the possibility for the implementation of broad inexpensive testing especially in areas where availability is restricted by economic or supply/demand issues.

## Conclusions

The purified recombinant Bst DNA polymerase and MM4-RT were used to develop fluorometric real time and colorimetric end-point RT-LAMP assays to detect the SARS-CoV-2 N gene. Colorimetric end-point RT-LAMP reactions without Tris-HCl or additives and with a low primer concentration were used successfully to detect SARS-CoV-2 in RNA isolated from saliva samples from infected individuals and to discriminate from negative samples. Fluorometric real time RT-LAMP represents a sensitive and efficient technique for quantifying viral loads, while colorimetric end-point RT-LAMP is an in-expensive and high throughput POC assay independent of sophisticated equipment.

## Supporting information

S1 Table Sequences of RT-LAMP primers for the detection of the nucleocapsid (N1) gene of SARS-CoV-2 and the human ribonuclease (RNase) P gene. (DOCX)

S1 Fig RT-LAMP primer sets evaluation. (A) Mapping of RT-LAMP N1 primer set that targets a region in the nucleocapsid (N) gene. Colored boxes represent the open reading frame (ORF) encoding N protein of SARS-CoV-2. (B) RT-LAMP reactions with N1 primer set. The figure shows the electrophoretic profile of the amplification reaction products in a 2% agarose gel. (C) RT-LAMP reactions with RP (human RNase P) primer set (internal control). The figure shows the electrophoretic profile of the amplification reaction products in a 2% agarose gel. F3: forward outer primer, FIP: forward inner primer, LF: loop forward primer, LB: loop backward primer, BIP: backward inner primer, B3: backward outer primer C+: 1x10^4^ copies of N1 *in vitro* transcript or human total RNA used as positive controls; NTC: non-template control; M: DNA molecular weight marker 1 Kb Plus DNA Ladder (Invitrogen).
(TIF)

S2 Fig Evaluation of induction conditions for recombinant *Bacillus stearothermophilus* DNA polymerase (Bst) expression. (A) Coomassie blue-stained 8% tricine-SDS-PAGE electrophoresis gel analysis of soluble fractions from triplicate experiments (1–3) evaluating different inducer concentrations (0.1, 0.5 and 1.0 mM IPTG) and induction temperatures (16 or 37°C) for Bst expression. (B) Coomassie blue-stained 8% tricine-SDS-PAGE electrophoresis gel analysis of soluble fractions from triplicate experiments (1–3) evaluating different growth medium (LB or TB) at 0.5 mM IPTG and 16°C for Bst expression. (C) Biomass [g/L] (y-axis) evaluated at different induction temperature (16 or 37°C) (x-axis) in bacterial cultures induced with 0.5 mM ITPG in LB medium. (D) Biomass [g/L] (y-axis) evaluated in different culture medium (LB or TB) (x-axis) in bacterial cultures induced with 0.5 mM ITPG at 16°C. (E) Biomass [g/L] (y-axis) evaluated with different inducer concentration (0, 0.1, 0.5 or 1 mM IPTG) (x-axis) in bacterial cultures grown at 16°C in LB medium. M: molecular weight marker; C+: previously purified Bst enzyme employed as control positive; C-: BL21(DE3)/pKJE7 untransformed culture used as negative control; NI: not induced bacterial culture; ns: no significant difference among treatments based on Dunn’s test at p < 0.05. Arrows indicate the expected size for Bst enzyme. Bars represent standard deviation.
(TIF)

S3 Fig Evaluation of induction conditions for recombinant reverse transcriptase (RT) expression. (A) Coomassie blue-stained 8% tricine-SDS-PAGE electrophoresis gel analysis of soluble fractions from triplicate experiments (1–3) evaluating different inducer concentrations (0.1, 0.5 and 1.0 mM IPTG) and induction temperatures (16 or 37°C) for RT expression. (B) Coomassie blue-stained 8% tricine-SDS-PAGE electrophoresis gel analysis of soluble fractions from triplicate experiments (1–3) evaluating different growth medium (LB or TB) at 0.5 mM IPTG and 16°C for RT expression. (C) Biomass [g/l] (y-axis) evaluated at different induction temperature (16 or 37°C) (x-axis) in bacterial cultures induced with 0.5 mM ITPG in LB medium. (D) Biomass [g/l] (y-axis) evaluated in different culture medium (LB or TB) (x-axis) in bacterial cultures induced with 0.5 mM ITPG at 16°C. (E) Biomass [g/l] (y-axis) evaluated with different inducer concentration (0, 0.1, 0.5 or 1 mM IPTG) (x-axis) in bacterial cultures grown at 16°C in LB medium. M: molecular weight marker; C+: previously purified RT enzyme employed as control positive; C-: BL21(DE3)/pKJE7 untransformed culture used as negative control; NI: not induced bacterial culture; ns: no significant difference among treatments based on Dunn’s test at p < 0.05. Arrows indicate the expected size for RT enzyme. Bars represent standard deviation.
(TIF)

S4 Fig Chromatographic profiles of the purification steps of Bst and RT by fast protein liquid chromatography (FPLC). (A) Chromatogram of Bst purification by Ni^+2^-IMAC. (B) Chromatogram of RT purification by Ni^+2^-IMAC. (C) Chromatogram of the desalting step of the Bst-containing fractions. (D) Chromatogram of the desalting step of the RT-containing fractions. (E) Chromatogram of the second purification step by heparin affinity chromatography for RT. (F) Chromatogram of the second purification step by cation exchange chromatography for RT. Values expressed in mAU are shown in purple (Bst) or green (RT). The dotted lines correspond to the concentration of the elution buffer used in each case: EB-AI (A), EB-BI (B), DB-A (C), DB-B (D), EB-AII (E), EB-B-II (F). Black arrows indicate the peaks of the chromatograms selected for the following purification steps.
(TIF)

S5 Fig Enzymatic activity assay of recombinant Bst and RT. Electrophoretic profile in 2% agarose gel of amplification products of LAMP assay or cDNA synthesis. (A) LAMP assay of N1 fragment with decreasing amounts of recombinant Bst using 437.5 pg (1) and 43.75 pg (2) of pDrive vector with N1 gene fragment as template. (B) cDNA synthesis with decreasing amounts of recombinant RT using 38 ng/μL of *in vitro* RP transcript. SSIII: SuperScript III Reverse Transcriptase (Invitrogen), used as control enzyme in cDNA synthesis. M: molecular weight marker 1 Kb Plus DNA Ladder (Invitrogen).
(TIF)

S6 Fig Effect of additives in colorimetric end-point RT-LAMP assay performance. (A) Colorimetric RT-LAMP reactions under optimized conditions using N1 primer set and 40 mM of guanidine hydrochloride (GuHCl) in the reaction buffer. The figure shows the colorimetric determination of each reaction (upper panel) and the electrophoretic profile of the amplification reaction products (lower panel). (B) Colorimetric RT-LAMP reactions under optimized conditions using N1 primer set in absence of GuHCl in the reaction buffer. The figure shows the colorimetric determination of each reaction (upper panel) and the electrophoretic profile of the amplification reaction products (lower panel). (C) Colorimetric RT-LAMP reactions under optimized conditions using N1 primer set and 0.8M of betaine in the reaction buffer. The figure shows the electrophoretic profile of the amplification reaction products. (D) Colorimetric RT-LAMP reactions under optimized conditions using N1 primer set in absence of betaine in the reaction buffer. The figure shows the electrophoretic profile of the amplification reaction products. C+: 1x10^4^ copies of N1 *in vitro* transcript used as positive control; NTC: non-template control; M: DNA molecular weight marker 1 Kb Plus DNA Ladder (Invitrogen).
(TIF)

S1 File SARS-CoV-2 detection by RT-qPCR (CDC and Berlin protocol-based methodologies) and colorimetric end-point RT-LAMP in saliva samples. (XLSX)
