# Supplementary material for: Starting from scratch: Step-by-step development of diagnostic tests for SARS-CoV-2 detection by RT-LAMP
Source: PLoS One. 2023 Jan 26;18(1):e0279681. doi: 10.1371/journal.pone.0279681 (PMC9879405; doi:10.1371/journal.pone.0279681)
Supplement: S1 Table — (DOCX) [file pone.0279681.s001.docx]

S1 Table. Sequences of RT-LAMP primers for the detection of the nucleocapsid (N1) of SARS-CoV-2 and the human ribonuclease (RNase) P gene.

| **Target** | **Name** | **Sequence (5’ – 3’)** | **Reference** |
| --- | --- | --- | --- |
| Nucleocapsid (N1) gene of SARS-CoV-2 | N1-FIP | CCACTGCGTTCTCCATTCTGGTAAATGCACCCCGCATTACG | Huang et al., 2018 |
|  | N1-BIP | CGCGATCAAAACAACGTCGGCCCTTGCCATGTTGAGTGAGA |  |
|  | N1-F3 | TGGACCCCAAAATCAGCG |  |
|  | N1-B3 | GCCTTGTCCTCGAGGGAAT |  |
|  | N1-LF | TGAATCTGAGGGTCCACCAAA |  |
|  | N1-LB | GGTTTACCCAATAATACTGCGTCTT |  |
| Human ribonuclease (Rnase) P gene | RP-FIP | GTGTGACCCTGAAGACTCGGTTTTAGCCACTGACTCGGATC | Curtis et al., 2018 |
|  | RP-BIP | CCTCCGTGATATGGCTCTTCGTTTTTTTCTTACATGGCTCTGGTC |  |
|  | RP-F3 | TTGATGAGCTGGAGCCA |  |
|  | RP-B3 | CACCCTCAATGCAGAGTC |  |
|  | RP-LF | ATGTGGATGGCTGAGTTGTT |  |
|  | RP-LB | CATGCTGAGTACTGGACCTC |  |
