# Supplementary figures and images for: Starting from scratch: Step-by-step development of diagnostic tests for SARS-CoV-2 detection by RT-LAMP
Source: PLoS One. 2023 Jan 26;18(1):e0279681. doi: 10.1371/journal.pone.0279681 (PMC9879405; doi:10.1371/journal.pone.0279681)

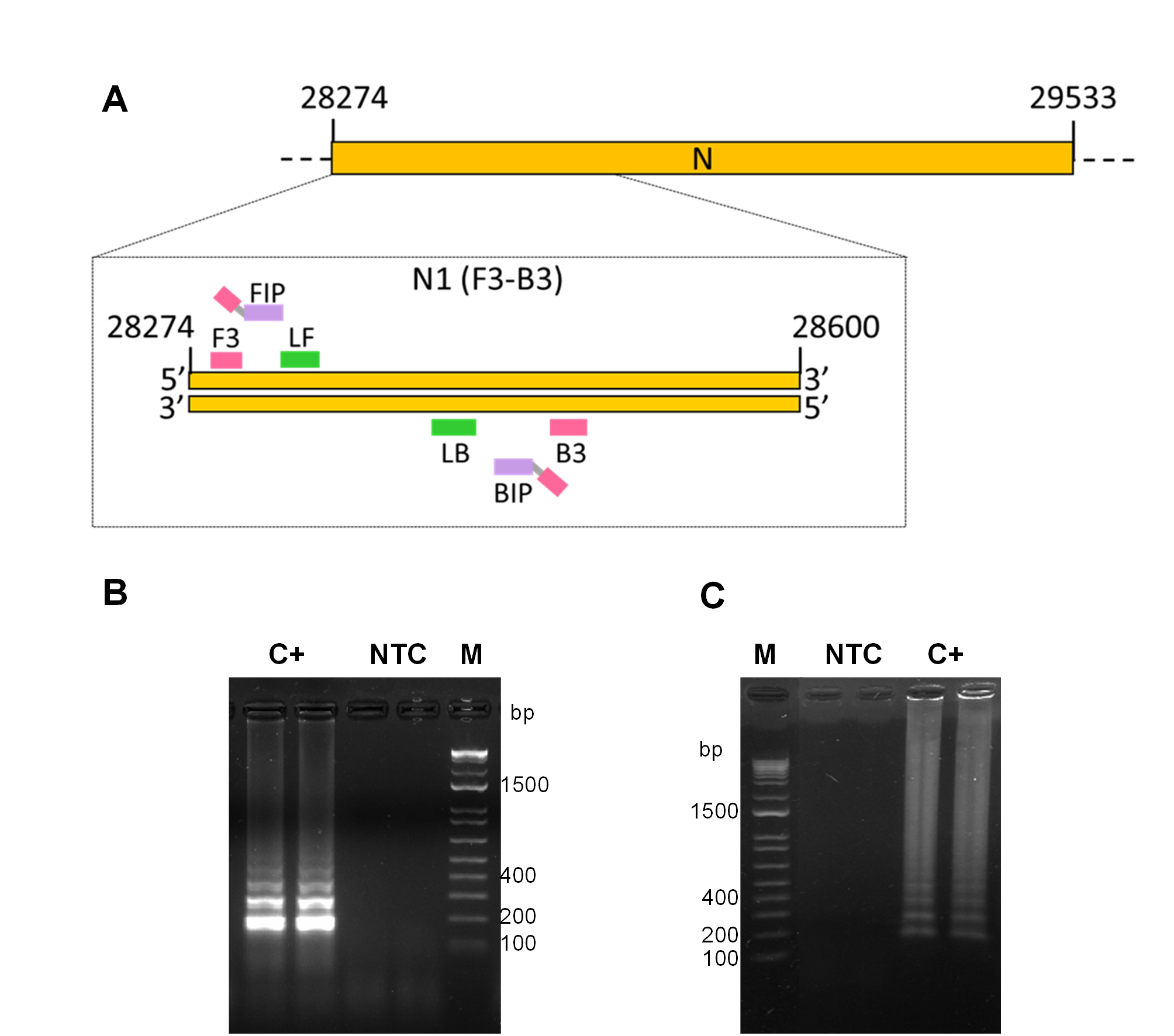

Supplement: S1 Fig — (A) Mapping of RT-LAMP N1 primer set that targets a region in the nucleocapsid (N) gene. Colored boxes represent the open reading frame (ORF) encoding N protein of SARS-CoV-2. (B) RT-LAMP reactions with N1 primer set. The figure shows the electrophoretic profile of the amplification reaction products in a 2% agarose gel. (C) RT-LAMP reactions with RP (human RNase P) primer set (internal control). The figure shows the electrophoretic profile of the amplification reaction products in a 2% agarose gel. F3: forward outer primer, FIP: forward inner primer, LF: loop forward primer, LB: loop backward primer, BIP: backward inner primer, B3: backward outer primer C+: 1x10^4^ copies of N1 in vitro transcript or human total RNA used as positive controls; NTC: non-template control; M: DNA molecular weight marker 1 Kb Plus DNA Ladder (Invitrogen). (TIF) [file pone.0279681.s002.tif]

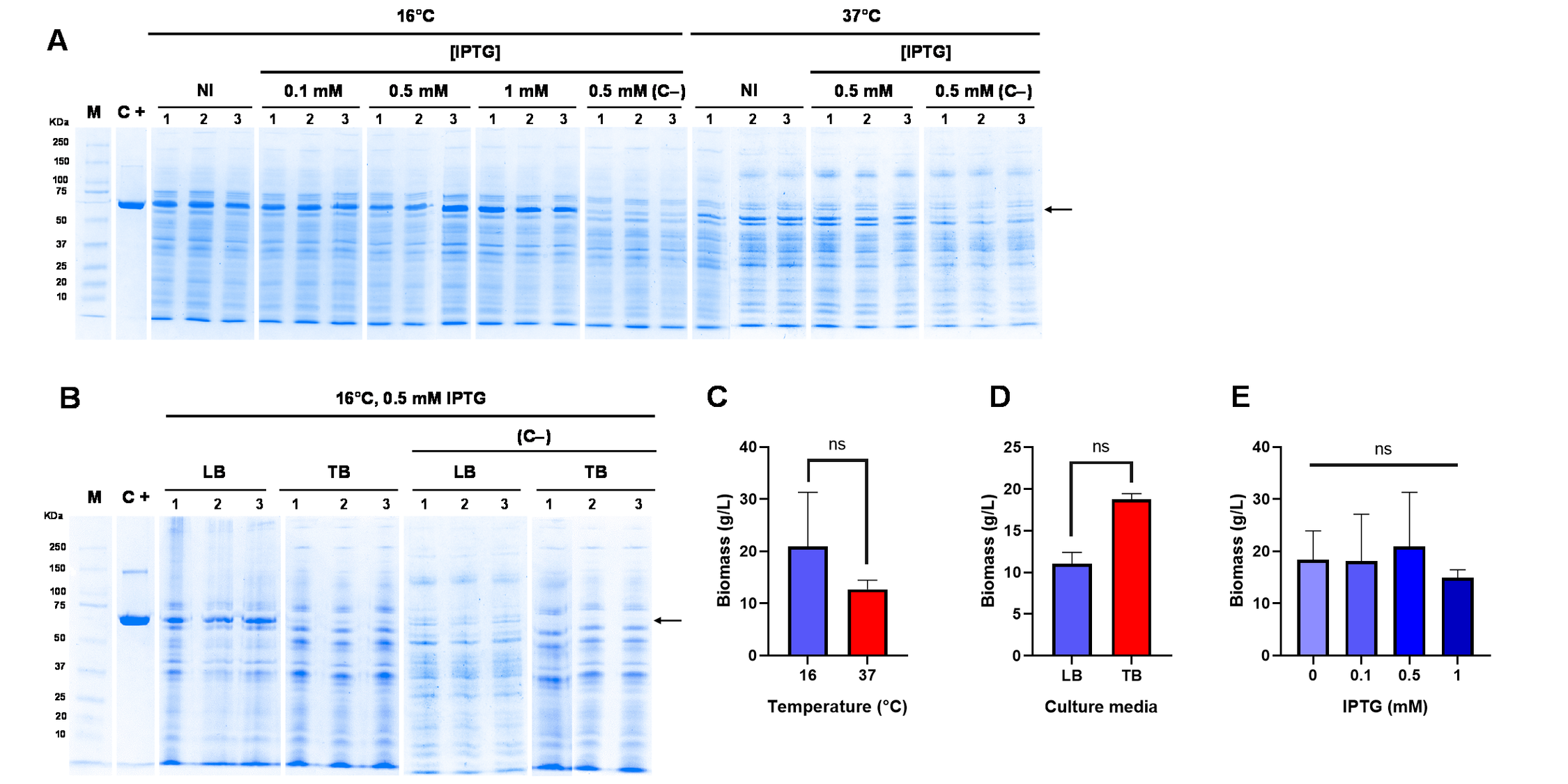

Supplement: S2 Fig — (A) Coomassie blue-stained 8% tricine-SDS-PAGE electrophoresis gel analysis of soluble fractions from triplicate experiments (1–3) evaluating different inducer concentrations (0.1, 0.5 and 1.0 mM IPTG) and induction temperatures (16 or 37°C) for Bst expression. (B) Coomassie blue-stained 8% tricine-SDS-PAGE electrophoresis gel analysis of soluble fractions from triplicate experiments (1–3) evaluating different growth medium (LB or TB) at 0.5 mM IPTG and 16°C for Bst expression. (C) Biomass [g/L] (y-axis) evaluated at different induction temperature (16 or 37°C) (x-axis) in bacterial cultures induced with 0.5 mM ITPG in LB medium. (D) Biomass [g/L] (y-axis) evaluated in different culture medium (LB or TB) (x-axis) in bacterial cultures induced with 0.5 mM ITPG at 16°C. (E) Biomass [g/L] (y-axis) evaluated with different inducer concentration (0, 0.1, 0.5 or 1 mM IPTG) (x-axis) in bacterial cultures grown at 16°C in LB medium. M: molecular weight marker; C+: previously purified Bst enzyme employed as control positive; C-: BL21(DE3)/pKJE7 untransformed culture used as negative control; NI: not induced bacterial culture; ns: no significant difference among treatments based on Dunn’s test at p < 0.05. Arrows indicate the expected size for Bst enzyme. Bars represent standard deviation. (TIF) [file pone.0279681.s003.tif]

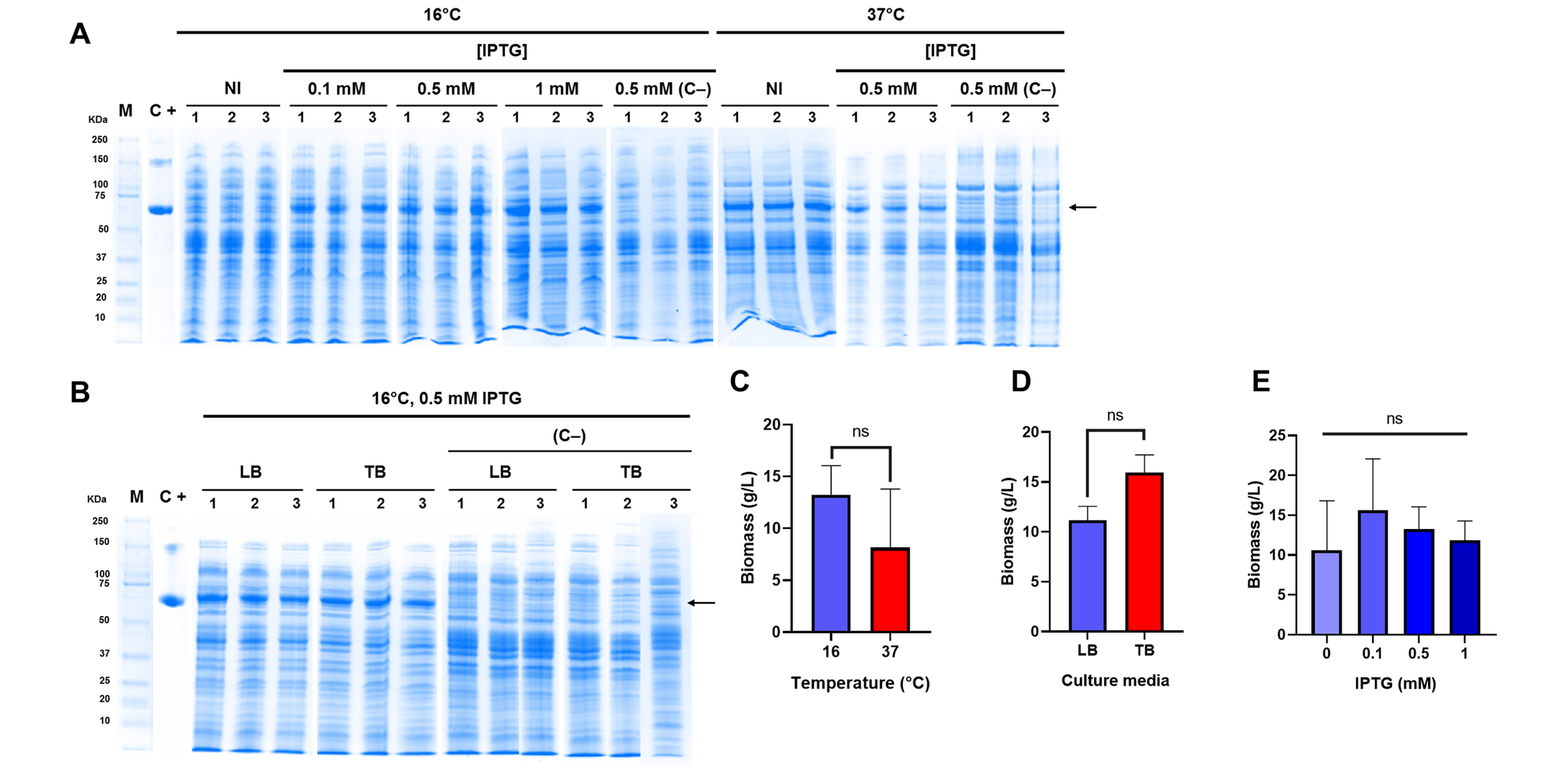

Supplement: S3 Fig — (A) Coomassie blue-stained 8% tricine-SDS-PAGE electrophoresis gel analysis of soluble fractions from triplicate experiments (1–3) evaluating different inducer concentrations (0.1, 0.5 and 1.0 mM IPTG) and induction temperatures (16 or 37°C) for RT expression. (B) Coomassie blue-stained 8% tricine-SDS-PAGE electrophoresis gel analysis of soluble fractions from triplicate experiments (1–3) evaluating different growth medium (LB or TB) at 0.5 mM IPTG and 16°C for RT expression. (C) Biomass [g/l] (y-axis) evaluated at different induction temperature (16 or 37°C) (x-axis) in bacterial cultures induced with 0.5 mM ITPG in LB medium. (D) Biomass [g/l] (y-axis) evaluated in different culture medium (LB or TB) (x-axis) in bacterial cultures induced with 0.5 mM ITPG at 16°C. (E) Biomass [g/l] (y-axis) evaluated with different inducer concentration (0, 0.1, 0.5 or 1 mM IPTG) (x-axis) in bacterial cultures grown at 16°C in LB medium. M: molecular weight marker; C+: previously purified RT enzyme employed as control positive; C-: BL21(DE3)/pKJE7 untransformed culture used as negative control; NI: not induced bacterial culture; ns: no significant difference among treatments based on Dunn’s test at p < 0.05. Arrows indicate the expected size for RT enzyme. Bars represent standard deviation. (TIF) [file pone.0279681.s004.tif]

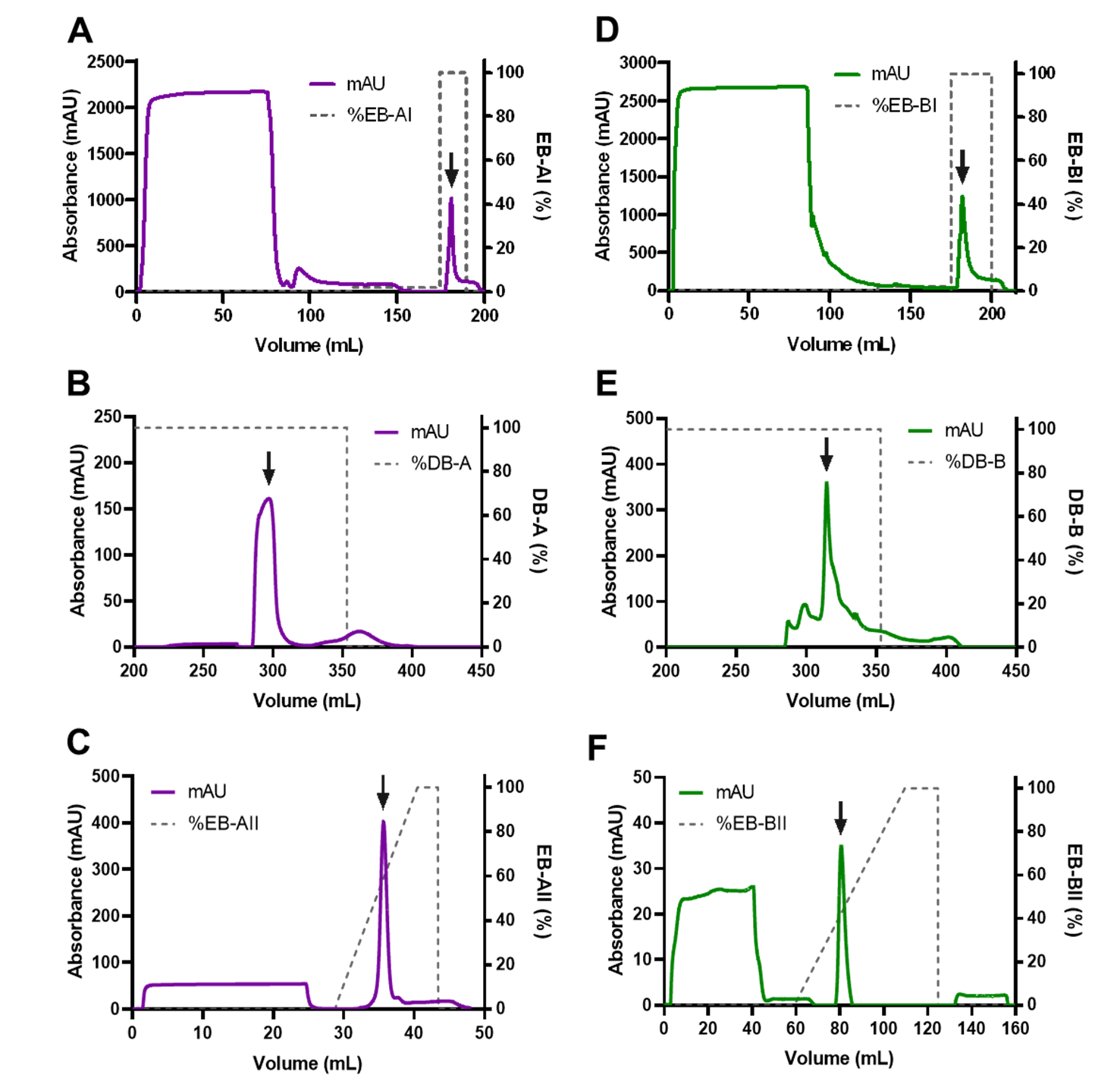

Supplement: S4 Fig — (A) Chromatogram of Bst purification by Ni+2-IMAC. (B) Chromatogram of RT purification by Ni+2-IMAC. (C) Chromatogram of the desalting step of the Bst-containing fractions. (D) Chromatogram of the desalting step of the RT-containing fractions. (E) Chromatogram of the second purification step by heparin affinity chromatography for RT. (F) Chromatogram of the second purification step by cation exchange chromatography for RT. Values expressed in mAU are shown in purple (Bst) or green (RT). The dotted lines correspond to the concentration of the elution buffer used in each case: EB-AI (A), EB-BI (B), DB-A (C), DB-B (D), EB-AII (E), EB-B-II (F). Black arrows indicate the peaks of the chromatograms selected for the following purification steps. (TIF) [file pone.0279681.s005.tif]

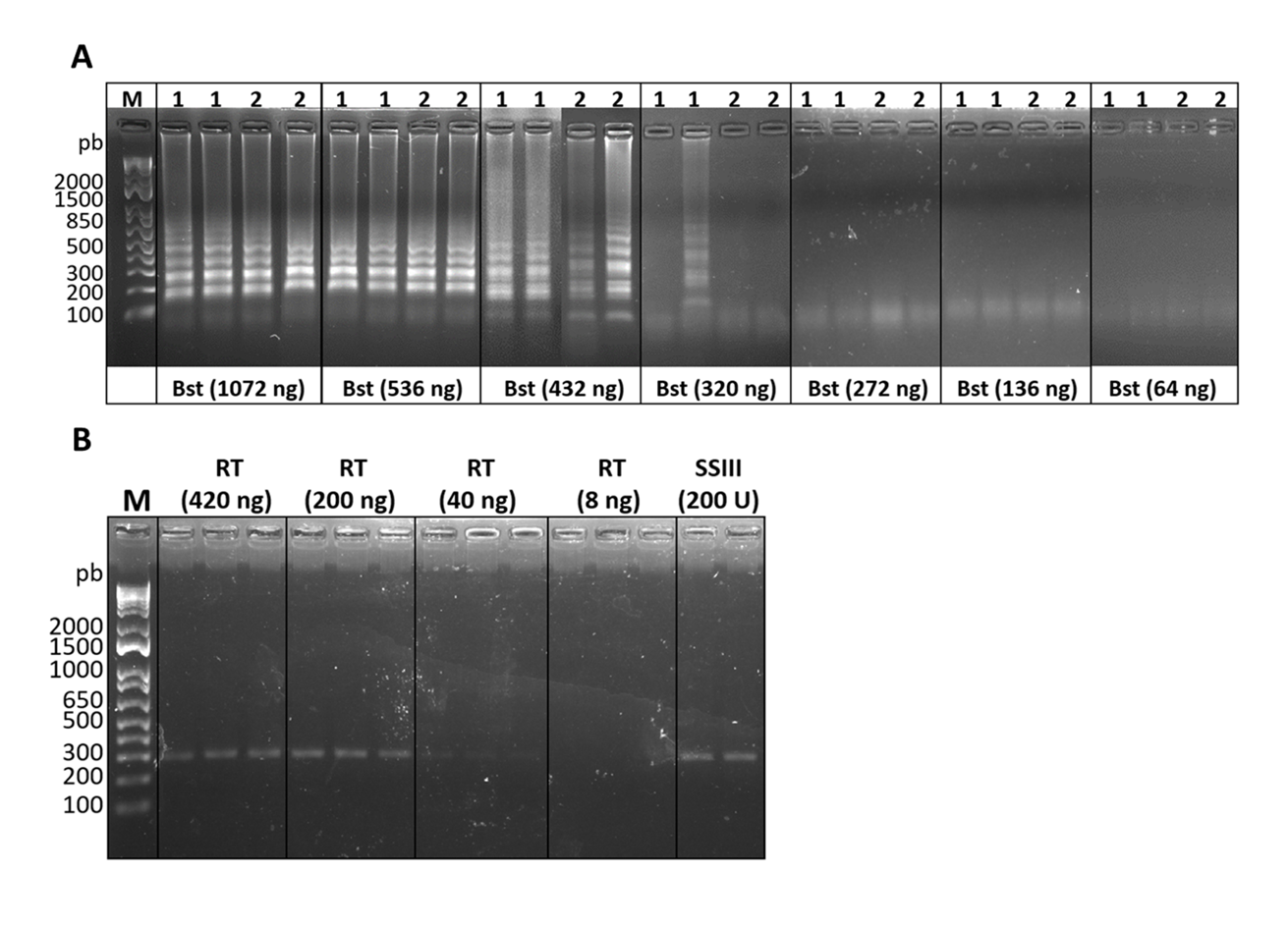

Supplement: S5 Fig — Electrophoretic profile in 2% agarose gel of amplification products of LAMP assay or cDNA synthesis. (A) LAMP assay of N1 fragment with decreasing amounts of recombinant Bst using 437.5 pg (1) and 43.75 pg (2) of pDrive vector with N1 gene fragment as template. (B) cDNA synthesis with decreasing amounts of recombinant RT using 38 ng/μL of in vitro RP transcript. SSIII: SuperScript III Reverse Transcriptase (Invitrogen), used as control enzyme in cDNA synthesis. M: molecular weight marker 1 Kb Plus DNA Ladder (Invitrogen). (TIF) [file pone.0279681.s006.tif]

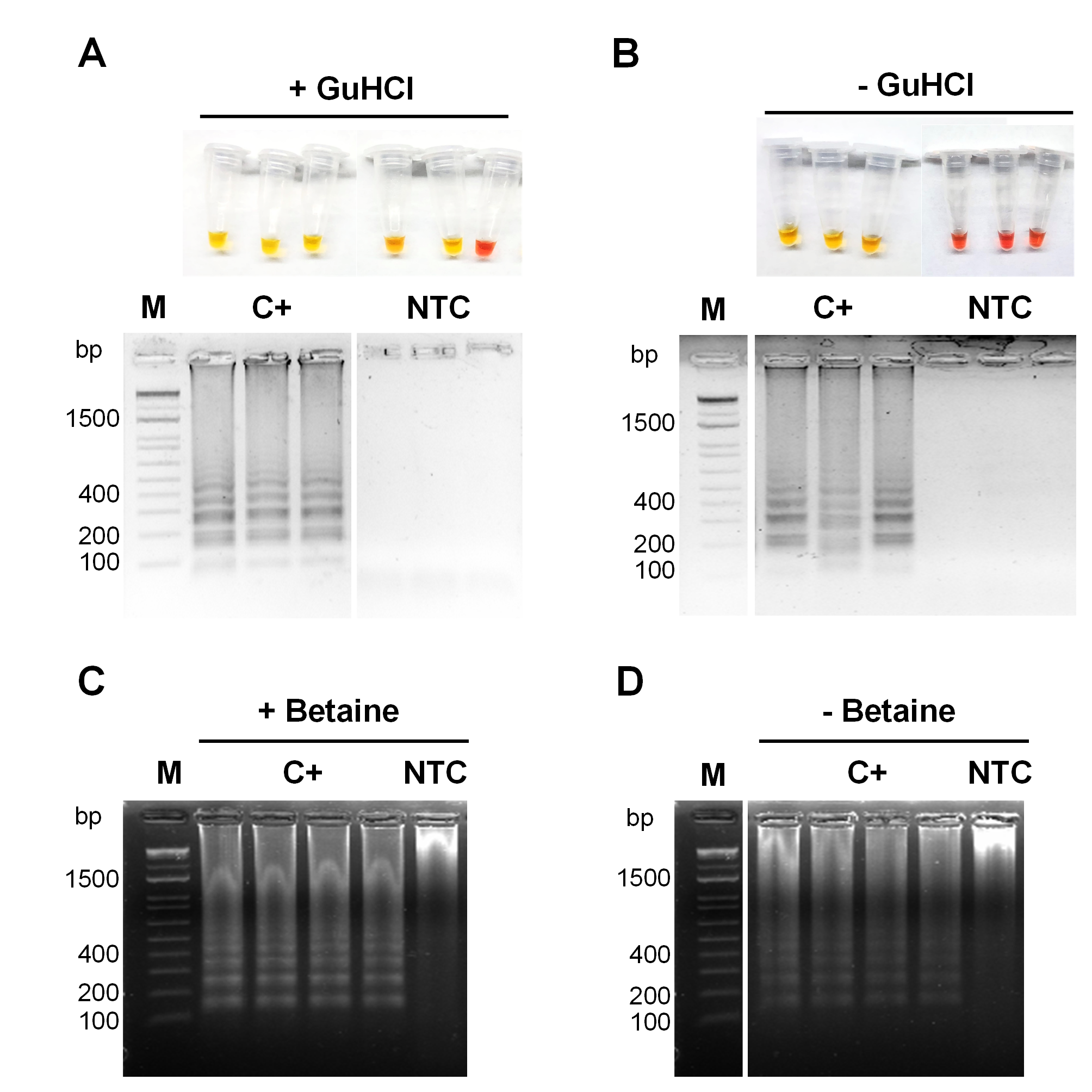

Supplement: S6 Fig — (A) Colorimetric RT-LAMP reactions under optimized conditions using N1 primer set and 40 mM of guanidine hydrochloride (GuHCl) in the reaction buffer. The figure shows the colorimetric determination of each reaction (upper panel) and the electrophoretic profile of the amplification reaction products (lower panel). (B) Colorimetric RT-LAMP reactions under optimized conditions using N1 primer set in absence of GuHCl in the reaction buffer. The figure shows the colorimetric determination of each reaction (upper panel) and the electrophoretic profile of the amplification reaction products (lower panel). (C) Colorimetric RT-LAMP reactions under optimized conditions using N1 primer set and 0.8M of betaine in the reaction buffer. The figure shows the electrophoretic profile of the amplification reaction products. (D) Colorimetric RT-LAMP reactions under optimized conditions using N1 primer set in absence of betaine in the reaction buffer. The figure shows the electrophoretic profile of the amplification reaction products. C+: 1x10^4^ copies of N1 in vitro transcript used as positive control; NTC: non-template control; M: DNA molecular weight marker 1 Kb Plus DNA Ladder (Invitrogen). (TIF) [file pone.0279681.s007.tif]
